# Mortality Associated With Occupational Exposure in Helsinki, Finland—A 24-Year Follow-up

**DOI:** 10.1097/JOM.0000000000002718

**Published:** 2022-10-06

**Authors:** Juuso Jalasto, Ritva Luukkonen, Ari Lindqvist, Arnulf Langhammer, Hannu Kankaanranta, Helena Backman, Eva Rönmark, Anssi Sovijärvi, Paula Kauppi, Päivi Piirilä

**Affiliations:** From the Department of Clinical Physiology, HUS Medical Diagnostic Center, Helsinki University Central Hospital and University of Helsinki, Helsinki, Finland (Dr Jalasto, Dr Sovijärvi, Dr Piirilä); Finnish Institute of Occupational Health, Helsinki, Finland (Dr Luukkonen); Department of Pulmonary Medicine, Heart and Lung Center, Helsinki University Hospital and Helsinki University, Helsinki, Finland (Dr Lindqvist, Dr Kauppi); HUNT Research Centre, Department of Public Health and Nursing, NTNU, Norwegian University of Science and Technology, Levanger, Norway (Dr Langhammer); Levanger Hospital, Nord-Trøndelag Hospital Trust, Levanger, Norway (Dr Langhammer); Krefting Research Centre, Institute of Medicine, Department of Internal Medicine and Clinical Nutrition, University of Gothenburg, Gothenburg, Sweden (Dr Kankaanranta); Department of Respiratory Medicine, Seinäjoki Central Hospital, Seinäjoki, Finland (Dr Kankaanranta); Tampere University Respiratory Research Group, Faculty of Medicine and Health Technology, Tampere University, Tampere, Finland (Dr Kankaanranta); and Department of Public Health and Clinical Medicine, Section of Sustainable Health, The OLIN Unit, Umeå University, Umeå, Sweden (Dr Backman, Dr Rönmark).

**Keywords:** asthma, COPD, Finland, prospective study, general population cohort, causes of death, airborne occupational exposure, job-exposure matrix, postal questionnaire, chronic lower airway disease

## Abstract

Our objective was to study mortality related to different obstructive lung diseases, occupational exposure, and their potential joint effect in a large, randomized population-based cohort.

**Methods:**

We divided the participants based on the answers to asthma and chronic obstructive pulmonary disease (COPD) diagnoses and occupational exposure and used a combined effects model and compared the results to no asthma or COPD with no occupational exposure.

**Results:**

High exposure had a hazards ratio (HR) of 1.34 (1.11–1.62) and asthma and COPD coexistence of 1.58 (1.10–2.27). The combined effects of intermediate exposure and coexistence had an HR of 2.20 (1.18–4.09), high exposure with coexistence of 1.94 (1.10–3.42) for overall mortality, and sub-HR for respiratory-related mortality of 3.21 (1.87–5.50).

**Conclusions:**

High occupational exposure increased overall but not respiratory-related mortality hazards, while coexisting asthma and COPD overall and respiratory-related hazards of mortality.

## Abstract 2

This paper studies the possible combined effect of occupational exposure and chronic airway disease to all-cause and respiratory related mortality in a longitudinal study setting. The results suggest that occupational airborne exposure increases mortality and is even more harmful for those with asthma and COPD coexistence.

**CME Learning Objectives**

After completing this enduring educational activity, the learner will be better able to:

- Discuss mortality related to obstructive lung diseases
- Outline the related occupational exposure and their potential joint effect in a large, randomized population-based cohort
- Compare the response to no asthma or COPD with occupational exposure

The evidence for the occupational burden of chronic obstructive pulmonary disease (COPD) and asthma has been substantial, and for coexisting asthma and COPD, it has been estimated to be at least 10%.^1^ Many of the epidemiological studies on the burden of occupational exposure have been performed in Europe and North America, and it has been thought that the burden might be even higher in countries with less regulation of work hygienic conditions.^2^

COPD prevalence and incidence have previously been shown to have a relationship to occupational exposure.^3,4^ In Finland, the COPD prevalence in the 1990s was approximately 4%^5^ and the prevalence of asthma approximately 7%.^6^ There was no large attributable amount of asthma due to occupational exposure (occupational sensitization or irritation) in Finland in the 1990s.^7^

Finland as a country has a unique system of statistical information, such as registry of deaths, gathered and maintained by Statistics Finland. In addition, the reimbursement of asthma medicines is tied to diagnosis of asthma supported by verified variable airway obstruction as shown by objective lung function tests. Similarly, COPD required objective spirometry results to qualify for medicine reimbursements. Before the mid-1990s, asthma was mostly diagnosed and treated by pulmonologists,^8^ and COPD was similarly handled by pulmonologists.

In epidemiological studies, airborne occupational exposures have been linked to the genesis of both COPD and asthma.^9–14^ Job-exposure matrix (JEM)–classified (occupational) exposure among occupations has previously shown manual work to have the biggest burden of exposure.^15^ In a previous cross-sectional study, we estimated the difference of occupational exposure among different occupational groups^6^ and found similar results.

Although mortality differences among different occupations have been reported earlier,^16–19^ few have studied mortality by diagnoses and occupational exposure in a longitudinal perspective with randomly sampled cohorts. The aim of the study was to estimate the all-cause and respiratory-related mortality associated with occupational exposure combined with data on self-reported physician diagnosed asthma or COPD in a large prospective cohort of 6062 participants with a 24-year follow-up.

## METHODS

The present data originate from the Helsinki part of the FinEsS study in Finland, Estonia, and Sweden, which began in 1996.

### Study Cohort

The study population was randomly selected by the Finnish national statistical service (Statistics Finland), aged 20–69 years, in the city of Helsinki in 1996. The sexes were randomized separately in 10 years age cohorts considering the overall distribution of sexes in the population. Postal questionnaires (*n* = 8000) were sent in 1996, 6062 (76%) responded to the questionnaire and 5271 (87%) had reported occupation title according to which we could assess an exposure rate based on a JEM.

### Mortality Data

The mortality data were obtained from Statistics Finland, using the participants’ personal identification code. We obtained the date and cause of death for all participants who died until December 31, 2019 given that they had successfully returned the questionnaire in 1996. All statistical data include the date of death of the participants alongside the underlying and potential contributing cause of death coded according to ICD-10.

### Definitions

The questionnaire questions used in this study and the occupation coding can be found in the supplementary materials (Supplementary Tables 1 and 2, http://links.lww.com/JOM/B216).

We defined occupational exposure as none, intermediate, and high depending on the occupational title and the occupational code assigned to the title previously. The assignment was done via a JEM from the ISCO (International Standard Classification of Occupations) version 1988 (ISCO-88) coding of the occupations. The assessment of the JEM values can be seen in Supplementary Table 3, http://links.lww.com/JOM/B216.

The time in principle occupation was obtained from the questionnaire and represents the time spent (in years) in the occupation, which was also used as the basis for the exposure figure.

From the answers to the postal questions, we were able to form groups by diagnosis, as well as smoking status. Educational level was obtained from the SEI occupational coding as it contains information for post-comprehensive education (in years) that each group needs in a Nordic country. Variables used in the analysis can be seen in Supplementary Table 4, http://links.lww.com/JOM/B216.

For this study, we combined the occupational exposure and diagnostic group values forming three combined variables (one for each diagnostic combination).

### Statistics

We analyzed the results first as separate effects for the diagnoses and occupational exposure and then combined them into a single variable and analyzed that in separate models. The separate models used no diagnose and no exposure as reference groups. The combined models used participants without self-reported asthma or COPD diagnosis and without exposure as the reference group. Cox proportional hazards models with follow-up of 24 years were used to compute hazards ratios (HRs) for the diagnoses and exposures. The full adjustment of the models included age, educational level, sex, and smoking status, excluding those without this information.

The respiratory disease–related models were computed with a competing-risks regression model, which is based on the Fine-Gray proportional sub-hazards model.^20^ The results are sub-hazards that tell of the hazards during the follow-up differentiating from the Cox regression hazards values. These were similarly adjusted for age, educational level, sex, and smoking status.

The Kaplan-Meier models included participants aged 50 years and older at the baseline of 1996. The survival analysis was done separately for each disease similarly to the regression models. The pairwise comparison used for testing the mean survival times was log-rank (Mantel-Cox) test, and each group was tested against the no diagnosis participants without occupational exposure. The age limit of 50 years was chosen to make the various groups more similar with regards to age.

All analyses were carried out using IBM SPSS Statistics Version 27 (IBM Corp, New York, NY) and StataCorp 2021, Stata Statistical Software: Release 17 (StataCorp LLC, College Station, TX). The statistical significance was set at 0.05.

## RESULTS

The difference in smoking habits, symptoms mortality, and education among the combined effects groups is shown in Table 1.

**TABLE 1:** Demographic Data, Smoking Habits, and Symptom Prevalence in Relation to Combined Effects

	No Diagnosis of Asthma or COPD			Asthma Alone			COPD Alone			Coexisting Asthma and COPD		
	No Exposure	Intermediate Exposure	High Exposure	No Exposure	Intermediate Exposure	High Exposure	No Exposure	Intermediate Exposure	High Exposure	No Exposure	Intermediate Exposure	High Exposure
*n*	2604	1388	705	153	86	48	73	43	31	24	20	16
Age (yrs), median in 1996 (IQR), y	43	43	45	43	43	43	48	50	54	59	58	61
Age (yrs) range in 1996	20–69	20–69	20–69	21–69	21–69	22–68	26–69	26–69	21–69	25–69	23–68	41–69
Median yrs in principle profession in 1996	15	15	15	16	17	12	20	21	20	22	28	28
Female	1648 (63)	765 (55)	232 (33)	111 (73)	55 (64)	24 (50)	48 (66)	24 (56)	9 (29)	12 (50)	10 (50)	4 (25)
Current smoking status in 1996												
Never-smoker	1372 (53)	635 (46)	240 (34)	83 (54)	44 (52)	19 (40)	16 (22)	14 (33)	7 (23)	11 (46)	6 (32)	1 (6)
Ex-smoker	436 (17)	246 (18)	126 (18)	31 (20)	14 (17)	8 (17)	14 (19)	8 (19)	2 (7)	6 (25)	3 (16)	7 (44)
Current smoker	788 (30)	506 (37)	337 (48)	39 (26)	26 (31)	20 (43)	43 (59)	20 (48)	22 (71)	7 (29)	10 (53)	8 (50)
Current daily smoking												
5 cig. per day	213 (27)	91 (18)	38 (11)	11 (28)	4 (15)	4 (20)	7 (16)	3 (15)	1 (5)	0 (0)	0 (0)	0 (0)
5–14 cig. per day	309 (39)	198 (39)	122 (36)	22 (56)	18 (69)	6 (30)	12 (28)	4 (20)	4 (18)	2 (29)	5 (50)	3 (38)
>14 cig. per day	266 (34)	217 (43)	177 (53)	6 (15)	4 (15)	10 (50)	24 (56)	13 (65)	17 (77)	5 (71)	5 (50)	5 (63)
Asthma medicine	19 (1)	18 (1)	10 (1)	108 (72)	56 (67)	32 (70)	9 (14)	7 (17)	3 (10)	20 (87)	15 (75)	14 (88)
Asthma symptoms	183 (7)	95 (7)	66 (10)	110 (72)	63 (74)	33 (69)	36 (51)	14 (34)	10 (32)	21 (88)	18 (90)	15 (94)
Allergic rhinitis	904 (35)	439 (32)	223 (32)	116 (76)	56 (67)	34 (72)	47 (65)	20 (47)	12 (41)	17 (71)	12 (63)	11 (73)
Chronic cough	405 (16)	228 (17)	152 (22)	56 (37)	36 (44)	15 (31)	52 (72)	24 (60)	18 (58)	17 (71)	13 (68)	13 (81)
Productive cough	542 (22)	315 (24)	231 (34)	69 (47)	43 (52)	23 (49)	56 (78)	34 (81)	27 (87)	20 (83)	15 (88)	13 (81)
Shortness of breath	253 (10)	160 (12)	102 (15)	54 (36)	36 (42)	18 (38)	37 (51)	20 (49)	19 (66)	17 (71)	14 (70)	14 (93)
Education level of occupation												
Low (<2 yrs)	721 (30)	470 (35)	338 (49)	32 (22)	28 (33)	26 (54)	20 (29)	18 (44)	18 (58)	8 (35)	7 (35)	5 (31)
Intermediate (2–5 yrs)	1079 (44)	844 (63)	350 (51)	70 (48)	57 (67)	22 (46)	34 (49)	22 (54)	13 (42)	9 (39)	13 (65)	11 (69)
High (>6 yrs)	668 (27)	29 (2)	2 (0)	43 (30)	0 (0)	0 (0)	16 (23)	1 (2)	0 (0)	6 (26)	0 (0)	0 (0)
Mortality												
All-cause mortality	366 (14)	240 (17)	179 (25)	22 (14)	14 (16)	13 (27)	20 (27)	11 (26)	15 (48)	10 (42)	11 (55)	11 (69)
Death associated with respiratory disease	67 (3)	56 (4)	37 (5)	7 (5)	3 (4)	3 (6)	6 (8)	5 (12)	6 (19)	3 (13)	6 (30)	9 (56)

Data are presented as *n* (%) unless otherwise stated.

COPD, chronic obstructive pulmonary disease; shortness of breath defined as having shortness of breath when walking on a flat ground with own age group; IQR, interquartile range; cig., cigarette.

At baseline, the most of the combined high exposure groups included more men, were older, had more current smokers, were more symptomatic, had lower education, and had higher all-cause mortality. Coexisting groups had the highest number of years in their principal occupation. The notable difference in this was the asthma alone groups, which had similar age and symptom tendencies among the different occupational exposures.

**FIGURE 1:**
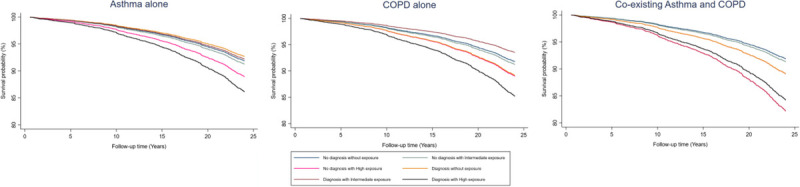
Combined effects all-cause mortality survival functions.

Table 2 contains the results of the all-cause mortality in the separate effects models and the combined effects models. Figure 1 shows the survival curves of the combined effects models for each disease group.

**TABLE 2:** Exposure and Diagnosis in Association to All-Cause Mortality Hazards Ratios

	All-Cause Mortality Hazards Models														
	Asthma Alone					COPD Alone					Coexisting Asthma and COPD				
	*n*	*n* Events (%)	HR	95%	CI	*n*	*n* Events (%)	HR	95%	CI	*n*	*n* Events (%)	HR	95%	CI
Adjusted separate effects model															
No diagnosis	4501	762 (17)	1	Reference		4501	762 (17)	1	Reference		4501	762 (17)	1	Reference	
Diagnosis	278	48 (17)	0.97	0.73	1.30	142	45 (32)	1.13	0.83	1.53	59	31 (53)	**1.58**	**1.10**	**2.27**
No exposure	2613	369 (14)	1	Reference		2538	368 (14)	1	Reference		2491	357 (14)	1	Reference	
Intermediate exposure	1428	250 (18)	1.07	0.91	1.27	1384	246 (18)	1.04	0.88	1.23	1363	247 (18)	1.08	0.91	1.28
High exposure	738	191 (26)	**1.39**	**1.15**	**1.68**	721	193 (26)	**1.35**	**1.11**	**1.63**	706	189 (26)	**1.34**	**1.11**	**1.62**
Adjusted combined effects model															
No diagnosis without exposure	2468	348 (14)	1	Reference		2468	348 (14)	1	Reference		2468	348 (14)	1	Reference	
No diagnosis with Intermediate exposure	1343	236 (18)	1.08	0.91	1.28	1343	236 (18)	1.07	0.90	1.27	1343	236 (18)	1.07	0.90	1.27
No diagnosis with high exposure	690	178 (26)	**1.36**	**1.12**	**1.65**	690	178 (26)	**1.34**	**1.10**	**1.62**	690	178 (26)	**1.35**	**1.11**	**1.63**
Disease, no exposure	145	21 (15)	0.90	0.59	1.37	70	20 (29)	1.31	0.87	1.99	23	9 (39)	1.34	0.71	2.56
Disease, intermediate exposure	85	14 (17)	0.96	0.54	1.71	41	10 (24)	0.79	0.40	1.56	20	11 (55)	**2.20**	**1.18**	**4.09**
Disease, high exposure	48	13 (27)	1.71	0.93	3.12	31	15 (48)	1.80	1.00	3.25	16	11 (69)	**1.94**	**1.10**	**3.42**

The models are adjusted for age, education, sex, and smoking status.

No diagnosis is defined as not having asthma or COPD, and event is defined as a reported by a death certificate.

Statistically significant results are bolded.

The *n* values presented here differ from the Table 1 values due to the education variable missing from some participants.

COPD, chronic obstructive pulmonary disease; CI, confidence interval.

In the separate effects model, high exposure group had a significant HR for all-cause mortality varying from 1.34 (1.11–1.62) to 1.38 (1.14–1.66) depending on the model. Only coexisting asthma and COPD had a significant HR (1.58; 95% confidence interval, 1.10–2.27).

In the combined model, only coexisting asthma and COPD showed significant results out of the disease groups, combined with either intermediate or high exposure, having HRs of 2.20 (1.18–4.09) and 1.94 (1.10–3.42), respectively.

For further analysis, we also computed a competing risks model for all respiratory causes using both the underlying and contributing causes of death. The results can be seen in Table 3.

**TABLE 3:** Exposure and Diagnose in Association to All-Cause Mortality Hazards Ratios

	Respiratory Disease–Related Mortality Sub-Hazards														
	Asthma Alone					COPD Alone					Coexisting Asthma and COPD				
	*n*	*n* Events (%)	HR	95%	CI	*n*	*n* Events (%)	HR	95%	CI	*n*	*n* Events (%)	HR	95%	CI
Adjusted separate effects model															
No diagnosis	4501	159 (4)	1	Reference		4501	159 (4)	1	Reference		4501	159 (4)	1	Reference	
Diagnosis	278	13 (5)	1.39	0.78	2.49	141	16 (11)	1.61	0.94	2.75	59	18 (31)	**3.21**	**1.87**	**5.50**
No exposure	2613	73 (3)	1	Reference		2538	72 (3)	1	Reference		2491	69 (3)	1	Reference	
Intermediate exposure	1428	59 (4)	1.25	0.87	1.78	1384	60 (4)	1.27	0.88	1.82	1363	62 (5)	1.33	0.93	1.92
High exposure	738	40 (5)	1.29	0.85	1.96	721	43 (6)	1.32	0.88	2.00	706	46 (7)	1.48	0.99	2.20
Adjusted combined effects model															
No diagnosis without exposure	2468	66 (3)	1	Reference		2468	66 (3)	1	Reference		2468	66 (3)	1	Reference	
No diagnosis with intermediate exposure	1343	56 (4)	1.30	0.90	1.88	1343	56 (4)	1.30	0.90	1.89	1343	56 (4)	1.30	0.90	1.87
No diagnosis with high exposure	690	37 (5)	1.31	0.85	2.01	690	37 (5)	1.31	0.85	2.01	690	37 (5)	1.31	0.85	2.01
Disease, no exposure	145	7 (5)	1.72	0.76	3.85	70	6 (9)	1.79	0.81	3.97	23	3 (13)	1.54	0.43	5.56
Disease, intermediate exposure	85	3 (4)	1.18	0.37	3.75	41	4 (10)	1.58	0.56	4.46	20	6 (30)	**3.85**	**1.38**	**10.7**
Disease, high exposure	48	3 (6)	2.01	0.59	6.87	31	6 (19)	2.41	0.95	6.15	16	9 (56)	**7.21**	**3.92**	**13.3**

The models are adjusted for age, education, sex, and smoking status.

No diagnosis is defined as not having asthma or COPD, and event is defined as a reported by a death certificate.

Statistically significant results are bolded.

COPD, chronic obstructive pulmonary disease: coexisting, self-reported physician made diagnosis of both asthma and COPD; CI, confidence interval.

These models were computed with the Fine-Gray model for competing risks. The results for the respiratory-related mortality are similar to all-cause mortality, although exposure alone does not give a significant sub-hazard. The result for coexisting asthma and COPD is significant both in the separate model as well as the combined effects model.

**FIGURE 2:**
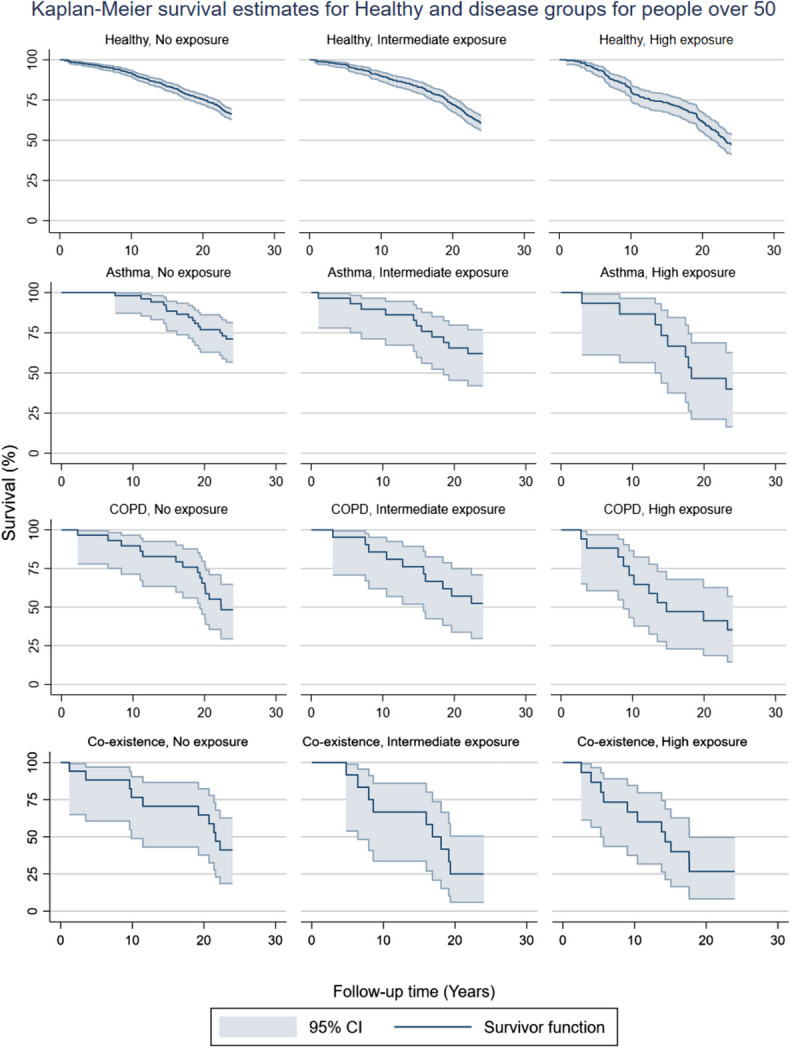
Survival curves in participants restricted to only those of 50 years and older.

The Kaplan-Meier survival model was used to compare the restricted mean survival times for participants who were at least 50 years old in 1996, using the combined effects variable. The results of this comparison can be seen in Supplementary Table 5, http://links.lww.com/JOM/B216. Figure 2 shows the survival curves of the model. The results are in line with the regression model with the lowest survival time found in the coexisting asthma and COPD with high exposure.

## DISCUSSION

The present study includes a 24-year prospective follow-up of a random population cohort collected in 1996 from Helsinki region in Finland. We found that JEM-assessed occupational exposure influences the overall mortality in all subjects. We also found that high exposure combined to self-reported coexisting physician diagnosed asthma and COPD had a higher hazard of overall mortality after a 24-year follow-up. Furthermore, we found that self-reported asthma and COPD coexistence had higher sub-HR of mortality due to respiratory causes.

Previous research^4^ has shown an association between occupational JEM-assessed exposure and more rapid progression of COPD in a smoking population in a cross-sectional analysis; however, they did not study mortality of their population. Occupation-related mortality has been previously explored in a limited setting inside certain manual occupations,^21^ which found occupational exposure to airborne pollution to increase mortality in a longitudinal study in COPD and never-smokers using a construction worker cohort. As far as we know, longitudinal analyses of occupational exposure on mortality in chronic obstructive airway diseases based on large general populations have not been published earlier. For the coexistence of asthma and COPD, relatively few studies exist and none that we know have analyzed the burden of airborne occupational exposure in long-term survival of coexisting asthma and COPD.

The attributable risk of occupational exposure to airway diseases is estimated to be somewhere approximately 10% to 20%.^1,2^ In Finland, there also exists a very strictly legally defined occupational asthma diagnosis group with its own governmentally mandated benefits. We were not able to recognize these as separate, entities and as such, they might be a minor part of the asthma or coexisting asthma and COPD groups. Registered cases of occupational asthma are relatively scarce in Finland,^7^ only accounting for a very small fraction of all asthma cases.

In an earlier study,^22^ we found that coexisting asthma and COPD cases have increased all-cause and respiratory mortality. The present results show a markedly high HR for the coexisting asthma and COPD associated with occupational exposure. The high occupational exposure to airborne particles accentuates the development of morbidity further. However, this finding is logical as particularly airborne particles have been shown to cause higher mortality in workers with professions as construction work^21^ or work-related COPD as a part of a mortality study concerning different professions.^23^

The effect of smoking on development of COPD is well known.^2,24,25^ Asthma can mostly be summarized as to having a reversible airway obstruction with corresponding variation of symptoms, whereas COPD can typically be characterized with a progressive mostly irreversible airflow limitation. Coexisting diagnoses of asthma and COPD can combine both the reversible nature and the progressive nature of the obstruction. The nature of both diseases is somewhat heterogenic and as such can also be the case of those with the coexisting diagnoses. The background causes for coexisting diagnosis are like to those of asthma or COPD with genetics, occupational exposure, as well as tobacco smoking being some of the main reasons. Chronic obstruction has also been observed in some asthma patients who have been resistant to asthma medication. Remodeling of the airways due to chronic inflammation has been thought to be an explanation.^26–28^ This can lead to a progressive obstructive disease, causing permanent lowering of lung function^29,30^ as seen in COPD. The Seinäjoki Adult Asthma Study^31^ also showed higher blood neutrophil levels and higher IL-6 levels, as well as more comorbidities alongside the reduced lung function in those with coexisting asthma and COPD. A recent study^32^ explored the trajectories of FEV1, FVC, and FEV1/FVC and found that the decline of lung function was the highest within the coexisting asthma and COPD group, although COPD had the highest lifelong exposure to tobacco. In the same study, roughly half of those in the asthma and COPD coexisting group had childhood asthma. This again underlies that the coexisting asthma and COPD group is heterogenic where the chronic inflammation and obstruction with partial reversibility can occur from varying causes. Although heterogenic, the coexisting group has been seen to be associated with more exacerbations than COPD alone.^33^ It is possible that particularly those with the asthma age of onset over 40 years are at danger of more strongly progressive disease with worse outcome.^34^

A recent meta-analysis, including 26 observational study articles,^35^ gave an estimate for the occurrence of coexisting asthma and COPD at approximately 2% of the population. These results seem to correspond well to our present study. In our previous study, we found that self-reported coexisting asthma and COPD had the highest all-cause and respiratory mortality.^22^ In the present study, we combined the diagnoses with an airborne exposure estimation, to see if there would be differences of survival among them and if the possible differences would depend on occupational exposure assessed by the main occupation. An involvement of exposure was found, especially in the mortality to asthma and COPD combination being one explanation to our previous results.^6^

The Finnish Social Security Institution is responsible for all medication reimbursements in Finland and finances its own research as well as uses the current research knowledge available in determining the guidelines in which medication costs are reimbursed in Finland. Because of this system, most diseases need a thorough examination to fulfill their criteria. This is also true for asthma as well as for COPD, both of which have needed measurement data (PEF surveillance or spirometry for asthma and spirometry for COPD), to be eligible for the reimbursement of medical costs. Because of this, most of the diagnoses self-reported by Finnish citizen have a modicum of reliance on not only symptoms but instrumental measurements as well. This medical compensation system has previously been somewhat problematic for those with COPD as they have not as easily been able to get reimbursement for their medication because of more strict diagnostic lung function criteria (FEV1 < 40% of predicted) compared with those with asthma diagnosis (the confirmation of reversibility of any level of obstruction), which may have affected the adherence to the treatment in those with COPD.

Occupational exposure can induce asthma or COPD or worsen an existing disease,^2,36^ and the present study points out the effect of occupational exposure to the hazards of all-cause and respiratory-related mortality. A previous occupation-specific research^21^ has indicated that a specific manual occupational exposure is linked with a higher all-cause mortality and mortality from COPD.

According to Finnish law, an employer must offer all its employees occupational health care. The purpose of occupational health care is to prevent work-related illnesses and accidents, promote the safety of the work environment, and maintain the health of workers throughout their working lives. High exposure alone had a significant hazard of mortality, and high exposure combined with asthma and COPD had even higher hazards as well as a sub-hazards of respiratory-related mortality. These results highlight the role of occupational legislation and occupational health care in prevention of premature or respiratory mortality. Further guidance and development are needed to minimize occupational respiratory exposure. Both exposure measurements and different kinds of methods to reduce any exposure are recommended.

We recommend that individuals with a preexisting pulmonary disease should be protected by further exposure by both PPE and other equipment if environmental exposure cannot be removed from the workplace air.

### Strengths

The main strength of our study was our initial cohort of 6062 persons from a well-responded postal questionnaire (76% response rate), from which, we could assess a JEM value for 5271 individuals at the baseline, although it is possible that some selection bias exists as the response rate was better in females than in men. Our study setting allowed us a long 24 years of follow-up time. We could also get smoking status and self-reported physician made diagnoses for asthma and COPD at the start. From the occupational categorizations made from the occupation title of the principal long-term occupation, we could form an education level for everyone based on the typical time of post-comprehensive education for the occupation category.

All deaths in Finland are recorded to the national statistics services (Statistics Finland) alongside the cause of death. In Finland, both a main and an immediate cause of death alongside up to four additional contributing factors to the cause of the death are assessed. This assessment is done either by an attending medical professional or in cases where the cause of death is not immediately clear enough an autopsy can be called, and it is done by a trained pathologist. The death certificates are also always validated by a forensic medical expert at Statistics Finland before entering them to the register. All underlying, immediate, and contributing causes of death are registered with a corresponding ICD-10 code.

High age of the participant is associated with COPD diagnose and asthma and COPD coexistence. To mitigate its effect in our results, we also analyzed separately participants who were 50 years or older at baseline with a Kaplan-Meier survival model, and the results in this analysis were similar to those in the Cox regression model.

The Finnish reimbursement system and the Finnish Social Security guidelines have likely caused that the given self-reported diagnoses have a good specificity, especially with the asthma diagnosis.

## LIMITATIONS

The occupational classifications for the 1996 study cohort were done from the postal questionnaire answers and reflect the way the occupations were understood in the mid-1990s (NYK and SEI coding). The original codes were done with the given occupational titles and may not always reflect on actual working conditions as occupations within the same title can vary. We used the JEM to assess a figure for airborne occupational exposure based on the main occupation title of the participant coded into ISCO-88. The original JEM used only three types of exposure (biological, mineral dust, and gas/fumes). These were then used to define none (no exposure in any group), intermediate (intermediate exposure in any group), and strong (strong exposure in any group) exposure groups. Specific named irritants and types of chemicals were not considered in the JEM, nor any measurements results were available, and the assignment of the tiers of exposure was not done at a specific level. Although the JEM would have allowed us to observe three different qualities of exposure, a decision was made to gather the three different exposures together as shown in Supplementary Table 3, http://links.lww.com/JOM/B216. This avoids some of the problems arising first from the ISCO-88 code approximation, as well as possible problems in the JEM using only a medium hierarchy occupational categorization of ISCO-88.

The exposure estimation was based on the main occupation at 1996 or earlier. Table 1 also shows the time spent in the main occupation, the median of which in most groups as in the excess of 20 years. Although some changes in the careers may have occurred during the 24-year follow-up, those at least 50 years old in 1996 are likely not to have changed their profession. The mortality results for those 50 years or older in 1996, as seen in Figure 2, are similar as the results of the whole patient material and suggest that the possible changes in professions during the follow-up would not be very important in this context. Unfortunately, it was not possible to afterward check their professional pathways.

As for the validity of the questions, the question on physician-diagnosed asthma has earlier been reported to have high specificity (94%) in a Swedish study,^37^ and although the question regarding COPD, which inquiries about chronic bronchitis or emphysema, does not fill the modern diagnostic criteria of COPD, it is in line for the period’s diagnostic practices.^38,39^ Based on this question with lower sensitivity, individuals with only chronic bronchitis can possibly have caused COPD alone to show inconclusive results in the analysis. On the other hand, the prevalence of COPD may be lower than in other studies of the time due to the strict reimbursement criteria for COPD and under recognition of COPD and its symptoms. For the COPD results specifically, these issues may cause mixed effects with some causing a negative bias (reimbursement criterion) and some positive (the lower prevalence overall on self-reported diagnosis). As there was a lack of spirometry data, we were not able to reevaluate these cases in the study.

In this study, the prevalence of the coexisting diagnosis in 1996 varied approximately 1% to 2% in males, and the high occupational exposure group has the highest prevalence. It is possible that this might have given some bias in our results as well, with the milder cases of both asthma and COPD being left undiagnosed and therefore strengthening the findings for the differences in mortality. For example, the overall COPD prevalence in a systematic worldwide review conducted in 2006 gave a 7% prevalence of COPD^40^ compared with the approximately 4% prevalence in our data (Table 3).

Although we looked at the whole group of respiratory mortality causes of death, it is possible that there has been some amount of underreporting of COPD in the cause of death diagnose codes as has been previously seen in Sweden,^41^ although the Finnish registry of deaths maintained by Statistic Finland regularly updates instructions on assigning causes of death.

## CONCLUSIONS

In this general population study, high occupational exposure alone increased overall but not respiratory-related mortality, whereas the coexistence of asthma and COPD combined with high occupational exposure carries the highest risk of both all-cause and respiratory mortality. People who work in occupations with high occupational exposure to airborne particles are at a higher risk of mortality and should be mindful of this risk, and care should be used in using protective elements, especially if they have an existing chronic lower airway disease.

## Supplementary Material
