# Supplementary material for: Mortality Associated With Occupational Exposure in Helsinki, Finland—A 24-Year Follow-up
Source: J Occup Environ Med. 2022 Oct 6;65(1):22–8. doi: 10.1097/JOM.0000000000002718 (PMC9835676; doi:10.1097/JOM.0000000000002718)
Supplement: Supplementary file 1 [file joem-65-022-s001.docx]

**Abstract**

Objectives: Our objective was to study mortality related to different obstructive lung diseases, occupational exposure, and their potential joint effect in a large, randomized population-based cohort.

Methods: We divided the participants based on the answers to asthma and COPD diagnoses and occupational exposure and used a combined effects model and compared the results to no asthma or COPD with no occupational exposure.

Results: High exposure had a hazard ratio (HR) 1.34 (1.11-1.62) and asthma and COPD coexistence 1.58 (1.10-2.27). The combined effects of intermediate exposure and coexistence had a HR 2.20 (1.18-4.09), high exposure with co-existence 1.94 (1.10-3.42) for overall mortality and sub-hazard ratio for respiratory related mortality sHR 3.21 (1.87-5.50).

Conclusions: High occupational exposure increased overall, but not respiratory related mortality hazard while co-existing asthma and COPD overall and respiratory related hazard of mortality.

**Keywords:**

asthma; COPD; Finland; prospective study; general population cohort; causes of death; airborne occupational exposure; job-exposure matrix; postal questionnaire; chronic lower airway disease;

# Introduction

The evidence for the occupational burden of COPD and asthma has been substantial, and for co-existing asthma and COPD it has been estimated to be at least 10% (1). Many of the epidemiological studies on the burden of occupational exposure have been performed in Europe and North America, and it has been thought that the burden might be even higher in countries with less regulation of work hygienic conditions (2).

COPD prevalence and incidence have previously been shown to have a relationship to occupational exposure (3, 4). In Finland, the COPD prevalence in the 1990s was around 4% (5) and the prevalence of asthma around 7% (6). There was no large attributable amount of asthma due to occupational exposure (occupational sensitization or irritation) in Finland in the 1990s (7).

Finland as a country has a unique system of statistical information, such as registry of deaths, gathered and maintained by Statistics Finland. In addition, the reimbursement of asthma medicines is tied to diagnosis of asthma supported by verified variable airway obstruction as shown by objective lung function tests. Similarly, COPD required objective spirometry results to qualify for medicine reimbursements. Before the mid-90s asthma was mostly diagnosed and treated by pulmonologists (8), and COPD was similarly handled by pulmonologists.

In epidemiological studies, airborne occupational exposures have been linked to the genesis of both COPD and asthma (9-14). Job Exposure Matrix (JEM) classified (occupational) exposure between occupations have previously shown manual work to have the biggest burden of exposure (15). In a previous cross-sectional study, we estimated the difference of occupational exposure between different occupational groups (6) and found similar results.

While mortality differences between different occupations have been reported earlier (16-19), few have studied mortality by diagnoses and occupational exposure in a longitudinal perspective with randomly sampled cohorts. The aim of the study was to estimate the all-cause and respiratory related mortality associated with occupational exposure combined with data on self-reported physician diagnosed asthma or COPD in a large prospective cohort of 6062 participants with a 24-year follow-up.

Methods

The present data originates from the Helsinki part of the FineEsS-study in Finland, Estonia and Sweden which began in 1996.

**Study Cohort:**

The study population was randomly selected by the Finnish national statistical service (Statistics Finland), aged 20–69 years, in the city of Helsinki in 1996. The sexes were randomized separately in 10 years age cohorts considering the overall distribution of sexes in the population. Postal questionnaires (n = 8000) were sent in 1996, 6062 (76%) responded to the questionnaire and 5271 (87%) had reported occupation title according to which we could assess an exposure rate based on a Job-exposure Matrix (JEM).

**Mortality data:**

The mortality data was obtained from Statistics Finland, using the participants personal identification code. We obtained the date and cause of death for all participants who died until 31.12.2019 given that they had successfully returned the questionnaire in 1996. All statistical data includes the date of death of the participants alongside of underlying and potential contributing cause of death coded according to ICD-10.

# Definitions:

The questionnaire questions used in this study and the occupation coding can be found in the supplementary materials (Supplementary Table 1 and 2, <http://links.lww.com/JOM/B216>).

We defined occupational exposure as none, intermediate and high depending on the occupational title and the occupational code assigned to the title previously. The assignment was done via a Job-exposure Matrix (JEM) from the ISCO (International Standard Classification of Occupations) version 1988 (ISCO-88) coding of the occupations. The assessment of the JEM values can be seen in Supplementary Table 3, <http://links.lww.com/JOM/B216>.

The time in principle occupation was obtained from the questionnaire and represents the time spent (in years) in the occupation which was also used as the basis for the exposure figure.

From the answers to the postal questions, we were able to form groups by diagnosis, as well as smoking status. Educational level was obtained from the SEI occupational coding as it contains information for post-comprehensive education (in years) that each group needs in a Nordic country. Variables used in the analysis can be seen in Supplementary Table 4, <http://links.lww.com/JOM/B216>.

For this study we combined the occupational exposure and diagnostic group values forming three combined variables (one for each diagnostic combination).

# Statistics

We analyzed the results first as separate effects for the diagnoses and occupational exposure and then combined them into a single variable and analyzed that in separate models. The separate models used no diagnose and no exposure as reference groups. The combined models used participants without self-reported asthma or COPD diagnosis and without exposure as the reference group. Cox proportional hazard models with follow-up of 24-years were used to compute hazard ratios for the diagnoses and exposures. The full adjustment of the models included age, educational level, sex, and smoking status excluding those without this information.

The respiratory disease related models were computed with a competing-risks regression model which is based on the Fine-Gray proportional sub-hazards model (20). The results are sub-hazards that tell of the hazard during the follow-up differentiating from the Cox regression hazard values. These were similarly adjusted for age, educational level, sex, and smoking status.

The Kaplan-Meier models included participants aged 50 and over at the baseline of 1996. The survival analysis was done separately for each disease similarly to the regression models. The pairwise comparison used for testing the mean survival times was Log-Rank (Mantel-Cox) test and each group was tested against the no diagnosis participants without occupational exposure. The age limit of 50 years was chosen to make the various groups more similar with regards to age.

All analyses were carried out using IBM SPSS Statistics versions 27 (IBM Corp, New York, NY, USA) and StataCorp. 2021. *Stata Statistical Software: Release 17*. College Station, TX: StataCorp LLC. Statistical significance was set at 0.05

# .Results

The difference in smoking habits, symptoms mortality and education between the combined effects groups is shown in Table 1.

At baseline, the most of the combined high exposure groups included more men, were older, had more current smokers, were more symptomatic, had lower education and had higher all-cause mortality. Co-existing groups had the highest number of years in their principal occupation. The notable difference in this was the asthma alone groups which had similar age and symptom tendencies between the different occupational exposures.

Table 2 contains the results of the all-cause mortality in the separate effects models and the combined effects models. Figure 1 shows the survival curves of the combined effects models for each disease group.

**Figure 1** Combined effects all-cause mortality survival functions

In the separate effects model High exposure group had a significant hazard ratio for all-cause mortality varying from 1.34 (1.11-1.62) to 1.38 (1.14-1.66) depending on the model. Only Co-existing Asthma and COPD had a significant hazard ratio (1.58, 95% CI 1.10-2.27).

In the combined model only Co-existing Asthma and COPD showed significant results out of the disease groups, combined with either intermediate or high exposure, having hazard ratios 2.20 (1.18-4.09) and 1.94 (1.10-3.42) respectively.

For further analysis, we also computed a competing risks model for all respiratory causes using both the underlying and contributing causes of death. The results can be seen in table 3.

These models were computed with the Fine-Gray model for competing risks. The results for the respiratory related mortality are similar to all-cause mortality though exposure alone does not give a significant sub-hazard. The result for Co-existing asthma and COPD is significant both in the separate model as well as the combined effects model.

The Kaplan-Meier survival model was used to compare the restricted mean survival times for participants who were at least 50-years old in 1996, using the combined effects variable. The results of this comparison can be seen in Supplementary Table 5, <http://links.lww.com/JOM/B216>. Figure 2 shows the survival curves of the model. The results are in line with the regression model with the lowest survival time found in the co-existing Asthma and COPD with high exposure.

**Figure 2.** Survival curves in participants restricted to only those of 50 years and older

# Discussion

The present study includes a 24-year prospective follow-up of a random population cohort collected in 1996 from Helsinki region in Finland. We found that JEM-assessed occupational exposure influences the overall mortality in all subjects. We also found that high exposure combined to self-reported co-existing physician diagnosed asthma and COPD had a higher hazard of overall mortality after a 24-year follow-up. Furthermore, we found that self-reported asthma and COPD co-existence had higher sub-hazard ratio of mortality due to respiratory causes.

Previous research (4) has shown an association between occupational JEM-assessed exposure and more rapid progression of COPD in a smoking population in a cross-sectional analysis, however, they did not study mortality of their population. Occupation related mortality has been previously explored in a limited setting inside certain manual occupations (21), which found occupational exposure to airborne pollution to increase mortality in a longitudinal study in COPD and never-smokers using a construction worker cohort. As far as we know, longitudinal analyses of occupational exposure on mortality in chronic obstructive airway diseases based on large general populations have not been published earlier. For the co-existence of asthma and COPD, relatively few studies exist and none that we know have analyzed the burden of airborne occupational exposure in long-term survival of co-existing asthma and COPD.

The attributable risk of occupational exposure to airway diseases is estimated to be somewhere around 10-20% (1, 2). In Finland, there also exists a very strictly legally defined occupational asthma diagnosis group with its own governmentally mandated benefits. We were not able to recognize these as separate entities and as such they might be a minor part of the asthma or co-existing asthma and COPD groups. Registered cases of occupational asthma are relatively scarce in Finland (7) only accounting for a very small fraction of all asthma cases.

In an earlier study (22), we found that co-existing asthma and COPD cases have increased all cause and respiratory mortality. The present results show a markedly high hazard ratio for the co-existing asthma and COPD associated with occupational exposure. The high occupational exposure to airborne particles accentuates the development of morbidity further. However, this finding is logical as particularly airborne particles have been shown to cause higher mortality in workers with professions as construction work (21) or work-related COPD as a part of a mortality study concerning different professions (23).

The effect of smoking on development of COPD is well known (2, 24, 25). Asthma can mostly be summarized as to having a reversible airway obstruction with corresponding variation of symptoms, while COPD can typically be characterized with a progressive mostly irreversible airflow limitation. Co-existing diagnoses of asthma and COPD can combine both the reversible nature as well as the progressive nature of the obstruction. The nature of both diseases is somewhat heterogenic, and as such can also be the case of those with the co-existing diagnoses. The background causes for co-existing diagnosis are like to those of asthma or COPD with genetics, occupational exposure as well as tobacco smoking being some of the main reasons. Chronic obstruction has also been observed in some asthma patients who have been resistant to asthma medication. Remodeling of the airways due to chronic inflammation has been thought to be an explanation (26-28). This can lead to a progressive obstructive disease, causing permanent lowering of lung function (29, 30) as seen in COPD. The Seinäjoki Adult Asthma Study (31) also showed higher blood neutrophil levels and higher IL-6 levels as well as more comorbidities alongside the reduced lung function in those with co-existing asthma and COPD. A recent study (32) explored the trajectories of FEV1, FVC and FEV1/FVC and found that the decline of lung function was the highest within the co-existing asthma and COPD group, though COPD had the highest lifelong exposure to tobacco. In the same study, roughly half of those in the asthma and COPD co-existing group had childhood asthma. This again underlies that the co-existing asthma and COPD group is heterogenic where the chronic inflammation and obstruction with partial reversibility can occur from varying causes. While heterogenic, the co-existing group has been seen to be associated with more exacerbations than COPD alone (33). It is possible that particularly those with the asthma age of onset over 40 years are at danger of more strongly progressive disease with worse outcome (34).

A recent meta-analysis, including 26 observational study articles (35), gave an estimate for the occurrence of co-existing asthma and COPD at around 2% of the population. These results seem to correspond well to our present study. In our previous study, we found that self-reported co-existing asthma and COPD had the highest all cause and respiratory mortality (22). In the present study, we combined the diagnoses with an airborne exposure estimation, to see if there would be differences of survival between them and if the possible differences would depend on occupational exposure assessed by the main occupation. An involvement of exposure was found especially in the mortality to asthma and COPD combination being one explanation to our previous results (6).

The Finnish Social Security Institution (KELA) is responsible for all medication reimbursements in Finland and finances its own research as well as uses the current research knowledge available in determining the guidelines in which medication costs are reimbursed in Finland. Due to this system most diseases need a thorough examination to fulfill their criteria. This is also true for asthma as well as for COPD, both of which have needed measurement data (PEF surveillance or spirometry for asthma and spirometry for COPD), to be eligible for the reimbursement of medical costs. Due to this, most of the diagnoses, self-reported by Finnish citizen have a modicum of reliance on not only symptoms but instrumental measurements, as well. This medical compensation system has previously been somewhat problematic for those with COPD as they have not as easily been able to get reimbursement for their medication because of more strict diagnostic lung function criteria (FEV1 < 40% of predicted) compared to those with asthma diagnosis (the confirmation of reversibility of any level of obstruction) which may have affected the adherence to the treatment in those with COPD.

Occupational exposure can induce asthma or COPD or worsen an existing disease (2, 36), and the present study points out the effect of occupational exposure to the hazard of all-cause and respiratory related mortality. A previous occupation specific research (21) has indicated that a specific manual occupational exposure is linked with a higher all-cause mortality and mortality from COPD.

According to Finnish law, an employer must offer all its employees occupational health care. The purpose of occupational health care is to prevent work-related illnesses and accidents, promote the safety of the work environment and maintain the health of workers throughout their working lives. High exposure alone had a significant hazard of mortality and high exposure combined with asthma and COPD had even higher hazard as well as a sub-hazard of respiratory related mortality. These results highlight the role of occupational legislation and occupational health care in prevention of premature or respiratory mortality. Further guidance and development are needed to minimize occupational respiratory exposure. Both exposure measurements and different kind of methods to reduce any exposure are recommended.

We recommend that individuals with a pre-existing pulmonary disease should be protected by further exposure by both PPE and other equipment if environmental exposure cannot be removed from the workplace air.

**Strengths:**

The main strength of our study was our initial cohort of 6062 persons from a well responded postal questionnaire (76% response rate), from which, we could assess a JEM value for 5271 individuals at the baseline, though it is possible that some selection bias exists as the response rate was better in females than in men. Our study setting allowed us a long 24-years of follow-up time. We could also get smoking status and self-reported physician made diagnoses for asthma and COPD at the start. From the occupational categorizations made from the occupation title of the principal long-term occupation, we could form an education level for everyone based on the typical time of post-comprehensive education for the occupation category.

All deaths in Finland are recorded to the national statistics services (Statistics Finland) alongside the cause of death. In Finland, both a main and an immediate cause of death alongside up to four additional contributing factors to the cause of the death are assessed. This assessment is done either by an attending medical professional or in cases where the cause of death is not immediately clear enough an autopsy can be called, and it is done by a trained pathologist. The death certificates are also always validated by a forensic medical expert at Statistics Finland before entering them to the register. All underlying, immediate, and contributing causes of death are registered with a corresponding ICD-10 code.

High age of the participant is associated with COPD diagnose and asthma and COPD co-existence. To mitigate its effect in our results we also analyzed separately participants who were 50 years old or older at baseline with a Kaplan-Meier survival model, and the results in this analysis were similar to those in the Cox regression model.

The Finnish reimbursement system and the Finnish Social Security guidelines have likely caused that the given self-reported diagnoses have a good specificity especially with the asthma diagnosis.

**Limitations**

The occupational classifications for the 1996 study cohort were done from the postal questionnaire answers and reflect the way the occupations were understood in the mid-1990s (NYK and SEI coding). The original codes were done with the given occupational titles and may not always reflect on actual working conditions as occupations within the same title can vary. We used the Job-exposure Matrix (JEM) to assess a figure for airborne occupational exposure based on the main occupation title of the participant coded into ISCO-88. The original job-exposure matrix (JEM) used only three types of exposure (Biological, Mineral dust and Gas/fumes). These were then used to define none (no exposure in any group), intermediate (intermediate exposure in any group) and strong (strong exposure in any group) exposure groups. Specific named irritants and types of chemicals were not considered in the JEM, nor any measurements results were available, and the assignment of the tiers of exposure was not done at a specific level. While the JEM would have allowed us to observe three different qualities of exposure, a decision was made to gather the three different exposures together as shown in Supplementary Table 3, <http://links.lww.com/JOM/B216>. This avoids some of the problems arising firstly from the ISCO-88 code approximation as well as possible problems in the JEM using only a medium hierarchy occupational categorization of ISCO-88.

The exposure estimation was based on the main occupation at 1996 or earlier. Table 1 also shows the time spent in the main occupation, the median of which in most groups as in the excess of 20 years. While some changes in the careers may have occurred during the 24-year follow-up, those at least 50-year-old in 1996 are likely not to have changed their profession. The mortality results for those 50-years or older in 1996, as seen in Figure 2, are similar as the results of the whole patient material and suggests that the possible changes in professions during the follow-up would not be very important in this context. Unfortunately, it was not possible to afterwards check their professional pathways.

As for the validity of the questions, the question on physician-diagnosed asthma has earlier been reported to have high specificity (94%) in a Swedish study (37), and though the question regarding COPD, which inquiries about chronic bronchitis or emphysema, does not fill the modern diagnostic criteria of COPD, it is in line for the time-period’s diagnostic practices (38, 39). Based on this question with lower sensitivity, individuals with only chronic bronchitis can possibly have caused COPD alone to show inconclusive results in the analysis. On the other hand, the prevalence of COPD may be lower than in other studies of the time due to the strict reimbursement criteria for COPD and under recognition of COPD and its symptoms. For the COPD results specifically, these issues may cause mixed effects with some causing a negative bias (reimbursement criterion) and some positive (the lower prevalence over-all on self-reported diagnosis). As there was a lack of spirometry data, we were not able to reevaluate these cases in the study.

In this study, the prevalence of the co-existing diagnosis in 1996 varied around 1-2% in males and the high occupational exposure group having the highest prevalence’s. It is possible that this might have given some bias in our results as well, with the milder cases of both asthma and COPD being left undiagnosed and therefore strengthening the findings for the differences in mortality. For example, the overall COPD prevalence in a systematic worldwide review conducted in 2006 gave a 7% prevalence of COPD (40) compared to the around 4% prevalence in our data (Table 3).

While we looked at the whole group of respiratory mortality causes of death, it is possible that there has been some amount of underreporting of COPD in the cause of death diagnose codes as has been previously seen in Sweden (41), though the Finnish registry of deaths maintained by Statistic Finland regularly updates instructions on assigning causes of death.

**Conclusions**

In this general population study, high occupational exposure alone increased overall but not respiratory related mortality, while the co-existence of asthma and COPD combined with high occupational exposure carries the highest risk of both all-cause and respiratory mortality. People who work in occupations with high occupational exposure to airborne particles are at a higher risk of mortality and should be mindful of this risk and care should be used in using protective elements, especially if they have an existing chronic lower airway disease.

# References:

1. Blanc PD, Annesi-Maesano I, Balmes JR, Cummings KJ, Fishwick D, Miedinger D et al. The Occupational Burden of Nonmalignant Respiratory Diseases. An Official American Thoracic Society and European Respiratory Society Statement. *Am J Respir Crit Care Med*. 2019;199(11):1312-1334. doi:10.1164/rccm.201904-0717ST
2. Global Initiative for Chronic Obstructive Lung Disease Global strategy for the diagnosis, management, and prevention of chronic obstructive pulmonary disease, Global Initiative for Chronic Obstructive Lung Disease; 2021 Report [Accessed 2021 November 25] Available from: <http://www.goldcopd.org/>
3. Mehta AJ, Miedinger D, Keidel D, Bettschart R, Bircher A, Bridevaux PO et al; SAPALDIA Team. Occupational exposure to dusts, gases, and fumes and incidence of chronic obstructive pulmonary disease in the Swiss Cohort Study on Air Pollution and Lung and Heart Diseases in Adults. Am J Respir Crit Care Med. 2012 Jun 15;185(12):1292-300. doi: 10.1164/rccm.201110-1917OC
4. Paulin LM, Diette GB, Blanc PD, Putcha N, Eisner MD, Kanner RE et al; SPIROMICS Research Group. Occupational exposures are associated with worse morbidity in patients with chronic obstructive pulmonary disease. Am J Respir Crit Care Med. 2015 Mar 1;191(5):557-65. doi: 10.1164/rccm.201408-1407OC
5. Vasankari TM, Impivaara O, Heliövaara M, Heistaro S, Liippo K, Puukka P et al. No increase in the prevalence of COPD in two decades. Eur Respir J. 2010 Oct;36(4):766-73. doi: 10.1183/09031936.00178109
6. Jalasto J, Lassmann-Klee P, Schyllert C, Luukkonen R, Meren M, Larsson M et al. Occupation, socioeconomic status and chronic obstructive respiratory diseases - The EpiLung study in Finland, Estonia and Sweden. Respir Med. 2022 Jan;191:106403. doi: 10.1016/j.rmed.2021.106403
7. Piipari R, Keskinen H. Agents causing occupational asthma in Finland in 1986-2002: cow epithelium bypassed by moulds from moisture-damaged buildings. Clin Exp Allergy. 2005 Dec;35(12):1632-7. doi: 10.1111/j.1365-2222.2005.02386.x
8. Haahtela T, Laitinen LA. Asthma programme in Finland 1994-2004. Report of a Working Group. Clin Exp Allergy. 1996 Jan;26 Suppl 1:i-ii, 1-24. doi: 10.1111/j.1365-2222.1996.tb02572.x
9. Pallasaho P, Kainu A, Sovijärvi A, Lindqvist A, Piirilä PL. Combined effect of smoking and occupational exposure to dusts, gases or fumes on the incidence of COPD. COPD J Chronic Obstr Pulm Dis. 2014;11(1):88-95. doi:10.3109/15412555.2013.830095
10. Eagan TM, Gulsvik A, Eide GE, Bakke PS. Occupational airborne exposure and the incidence of respiratory symptoms and asthma. Am J Respir Crit Care Med. 2002 Oct 1;166(7):933-8. doi: 10.1164/rccm.200203-238OC
11. Venables KM, Chan-Yeung M. Occupational asthma. Lancet. 1997 May 17;349(9063):1465-9. doi: 10.1016/S0140-6736(96)07219-4
12. Schyllert C, Rönmark E, Andersson M, Hedlund U, Lundbäck B, Hedman L et al. Occupational exposure to chemicals drives the increased risk of asthma and rhinitis observed for exposure to vapours, gas, dust and fumes: a cross-sectional population-based study. Occup Environ Med. 2016 Oct;73(10):663-9. doi: 10.1136/oemed-2016-103595
13. Hagstad S, Backman H, Bjerg A, Ekerljung L, Ye X, Hedman L et al. Prevalence and risk factors of COPD among never-smokers in two areas of Sweden - Occupational exposure to gas, dust or fumes is an important risk factor. Respir Med. 2015 Nov;109(11):1439-45. doi: 10.1016/j.rmed.2015.09.012
14. Salvi SS, Barnes PJ. Chronic obstructive pulmonary disease in non-smokers. Lancet. 2009 Aug 29;374(9691):733-43. doi: 10.1016/S0140-6736(09)61303-9
15. Schyllert C, Andersson M, Hedman L, Ekström M, Backman H, Lindberg A et al. Job titles classified into socioeconomic and occupational groups identify subjects with increased risk for respiratory symptoms independent of occupational exposure to vapour, gas, dust, or fumes. Eur Clin Respir J. 2018 May 15;5(1):1468715. doi: 10.1080/20018525.2018.1468715
16. Paglione L, Angelici L, Davoli M, Agabiti N, Cesaroni G. Mortality inequalities by occupational status and type of job in men and women: results from the Rome Longitudinal Study. BMJ Open. 2020 Jun 3;10(6):e033776. doi: 10.1136/bmjopen-2019-033776
17. Lee HE, Kim HR, Chung YK, Kang SK, Kim EA. Mortality rates by occupation in Korea: a nationwide, 13-year follow-up study. Occup Environ Med. 2016 May;73(5):329-35. doi: 10.1136/oemed-2015-103192
18. Niedhammer I, Bourgkard E, Chau N; Lorhandicap Study Group. Occupational and behavioural factors in the explanation of social inequalities in premature and total mortality: a 12.5-year follow-up in the Lorhandicap study. Eur J Epidemiol. 2011 Jan;26(1):1-12. doi: 10.1007/s10654-010-9506-9
19. Dodd KE, Wood J, Mazurek JM. Mortality Among Persons with Both Asthma and Chronic Obstructive Pulmonary Disease Aged ≥25 Years, by Industry and Occupation - United States, 1999-2016. MMWR Morb Mortal Wkly Rep. 2020 Jun 5;69(22):670-679. doi: 10.15585/mmwr.mm6922a3
20. J.P. Fine, and R.J. Gray, *A proportional hazards model for the subdistribution of a competing risk*. J. Am. Stat. Assoc. 94 (1999), pp. 496–509. doi: 10.1080/01621459.1999.10474144
21. Torén K, Järvholm B. Effect of occupational exposure to vapors, gases, dusts, and fumes on COPD mortality risk among Swedish construction workers: a longitudinal cohort study. Chest. 2014 May;145(5):992-997. doi: 10.1378/chest.13-1429
22. Jalasto J, Kauppi P, Luukkonen R, Lindqvist A, Langhammer A, Kankaanranta H et al. Self-Reported Physician Diagnosed Asthma with COPD is Associated with Higher Mortality than Self-Reported Asthma or COPD Alone - A Prospective 24-Year Study in the Population of Helsinki, Finland. COPD. 2022;19(1):226-235. doi: 10.1080/15412555.2022.2061935
23. Järvholm B, Reuterwall C, Bystedt J. Mortality attributable to occupational exposure in Sweden. Scand J Work Environ Health. 2013 Jan;39(1):106-11. doi: 10.5271/sjweh.3284
24. Sin DD, Miravitlles M, Mannino DM, Soriano JB, Price D, Celli BR et al. What is asthma-COPD overlap syndrome? Towards a consensus definition from a round table discussion. Eur Respir J. 2016 Sep;48(3):664-73. doi: 10.1183/13993003.00436-2016
25. Sears MR. Smoking, asthma, chronic airflow obstruction and COPD. Eur Respir J. 2015 Mar;45(3):586-8. doi: 10.1183/09031936.00231414
26. Pascual RM, Peters SP. The irreversible component of persistent asthma. J Allergy Clin Immunol. 2009 Nov;124(5):883-90; quiz 891-2. doi: 10.1016/j.jaci.2009.09.047
27. Fish JE, Peters SP. Airway remodeling and persistent airway obstruction in asthma. J Allergy Clin Immunol. 1999 Sep;104(3 Pt 1):509-16. doi: 10.1016/s0091-6749(99)70315-5
28. Lazaar AL, Panettieri RA Jr. Airway smooth muscle: a modulator of airway remodeling in asthma. J Allergy Clin Immunol. 2005 Sep;116(3):488-95; quiz 496. doi: 10.1016/j.jaci.2005.06.030
29. Backman H, Jansson SA, Stridsman C, Muellerova H, Wurst K, Hedman L et al. Chronic airway obstruction in a population-based adult asthma cohort: Prevalence, incidence and prognostic factors. Respir Med. 2018 May;138:115-122. doi: 10.1016/j.rmed.2018.03.036
30. Lange P, Parner J, Vestbo J, Schnohr P, Jensen G. A 15-year follow-up study of ventilatory function in adults with asthma. N Engl J Med. 1998 Oct 22;339(17):1194-200. doi: 10.1056/NEJM199810223391703
31. Tommola M, Ilmarinen P, Tuomisto LE, Lehtimäki L, Haanpää J, Niemelä O et al. Differences between asthma-COPD overlap syndrome and adult-onset asthma. Eur Respir J. 2017 May 1;49(5):1602383. doi: 10.1183/13993003.02383-2016
32. Marcon A, Locatelli F, Dharmage SC, Svanes C, Heinrich J, Leynaert B et al; Ageing Lungs in European Cohorts (ALEC) study. The coexistence of asthma and COPD: risk factors, clinical history and lung function trajectories. Eur Respir J. 2021 Nov 25;58(5):2004656. doi: 10.1183/13993003.04656-2020.
33. Zhou XL, Zhao LY. Comparison of clinical features and outcomes for asthma-COPD overlap syndrome vs. COPD patients: a systematic review and meta-analysis. Eur Rev Med Pharmacol Sci. 2021 Feb;25(3):1495-1510. doi: 10.26355/eurrev_202102_24857
34. Lange P, Çolak Y, Ingebrigtsen TS, Vestbo J, Marott JL. Long-term prognosis of asthma, chronic obstructive pulmonary disease, and asthma-chronic obstructive pulmonary disease overlap in the Copenhagen City Heart study: a prospective population-based analysis. Lancet Respir Med. 2016 Jun;4(6):454-62. doi: 10.1016/S2213-2600(16)00098-9
35. Hosseini M, Almasi-Hashiani A, Sepidarkish M, Maroufizadeh S. Global prevalence of asthma-COPD overlap (ACO) in the general population: a systematic review and meta-analysis. Respir Res. 2019 Oct 23;20(1):229. doi: 10.1186/s12931-019-1198-4
36. Global Initiative for Asthma Global strategy for asthma management and prevention, Global Initiative for Asthma; 2020 Report [Accessed 2021 November 25] Available from: <https://www.ginasthma.org/>
37. Torén K, Brisman J, Järvholm B. Asthma, and asthma-like symptoms in adults assessed by questionnaires. A literature review. Chest. 1993 Aug;104(2):600-8. doi: 10.1378/chest.104.2.600
38. Flenley DC. Chronic obstructive pulmonary disease. Dis Mon. 1988 Sep;34(9):537-99. doi: 10.1016/0011-5029(88)90015-6
39. Snider GL. Distinguishing among asthma, chronic bronchitis, and emphysema. Chest. 1985 Jan;87(1 Suppl):35S-39S. doi: 10.1378/chest.87.1.35s
40. Halbert RJ, Natoli JL, Gano A, Badamgarav E, Buist AS, Mannino DM. Global burden of COPD: systematic review and meta-analysis. Eur Respir J. 2006 Sep;28(3):523-32. doi: 10.1183/09031936.06.00124605
41. Lindberg A, Lindberg L, Sawalha S, Nilsson U, Stridsman C, Lundbäck B et al. Large underreporting of COPD as cause of death-results from a population-based cohort study. Respir Med. 2021 Sep;186:106518. doi: 10.1016/j.rmed.2021.106518

Figure/caption list:

1. **Figure 1.** Combined effects all-cause mortality survival functions
2. **Figure 2.** Survival curves in participants restricted to only those of 50 years and older
