# Supplementary material for: Mortality Associated With Occupational Exposure in Helsinki, Finland—A 24-Year Follow-up
Source: J Occup Environ Med. 2022 Oct 6;65(1):22–8. doi: 10.1097/JOM.0000000000002718 (PMC9835676; doi:10.1097/JOM.0000000000002718)
Supplement: Supplementary file 2 [file joem-65-022-s002.docx]

# Supplementary data

Supplementary table 5 gathers all the relevant postal questionnaire questions from 1996 used in this study. These questions seek to establish and describe the cohort at the baseline and the differences they might have to begin with. They also display the main questions where certain variables used in the analysis of the data could be formed such as smoking habit and occupation title.

**Postal questionnaire:**

The postal questionnaire used was based on the OLIN (Obstructive Lung Diseases in Northern Sweden) research project questionnaire (Lundbäck 1991, Pallasaho 1999) which was a further development of the British Medical Research Council (BMJ 1960) and Tucson questionnaires (Lebowitz 1975).

Lundbäck B, Nyström L, Rosenhall L, Stjernberg N. Obstructive lung disease in northern Sweden: respiratory symptoms assessed in a postal survey. Eur Respir J. 1991;4(3):257‐266.

Medical Research Council Committee on the aetiology of chronic bronchitis. Standardised questionnaires on respiratory symptoms. BMJ 1960;2:1665.

Lebowitz M, Knudson R, Burrows B. The Tucson epidemiology study of chronic obstructive lung disease. I: Methodology and prevalence of disease. Am J Epidemiol 1975; 102:137–152

Pallasaho P, Lundbäck B, Läspä SL, Jönsson E, Kotaniemi J, Sovijärvi AR, Laitinen LA. Increasing prevalence of asthma but not of chronic bronchitis in Finland? Report from the FinEsS-Helsinki Study. Respir Med. 1999 Nov;93(11):798-809. doi: 10.1016/s0954-6111(99)90265-2

**Supplementary table 5.** FinEsS-questionnaire questions translated in English

| Questions related to asthma: | *Asthma diagnosis by a physician* | Have you been diagnosed as having asthma by a physician | Yes/No |
| --- | --- | --- | --- |
|  | *Asthma medicine use* | Do you use any asthma medicine? (Medicine used was not defined) | Yes/No |
|  | *Asthma symptoms* | Have you, during the last 12 months, had intermittent attacks or periodic breathlessness, with or without cough or wheezing/whistling in your chest? | Yes/No |
|  | *Allergic rhinitis* | Have you ever had allergic rhinitis (e.g., hay fever) or allergic eye condition? | Yes/No |
| Questions related to COPD: | *COPD diagnosis by a physician* | Have you been diagnosed as having chronic bronchitis or emphysema by a physician? | Yes/No |
|  | *Chronic cough* | Have you had longstanding cough during the last year? | Yes/No |
|  | *Sputum production* | Do you usually have phlegm when coughing or do you have phlegm in your chest, which is difficult to bring up? | Yes/No |
| Question related to physical status: | *Shortness of breath (SOB***)** | Do you have shortness of breath or do you have to walk slower due to shortness of breath when you are walking on even ground with your age group at a normal pace | Yes/No |
| Smoking habit: | *Current smoker* | Do you smoke currently? (answer yes even if you have quit in the past 12 months) | Yes/No |
|  | *Cigars per day* | How many cigarettes do you smoked on per day? (answer only if you answered Yes to do you smoke currently) | less than 5 5-14 15 or more |
|  | *Ex-smoker* | Have you been a smoker before, but quit smoking over 12 months ago? (answer only if you answered No to the question of current smoking) | Yes/No |
| Questions related to occupation: | *Occupation title* | What has been your main work/occupation? | Title of occupation |

The original questionnaire had all the questions in both Finnish and Swedish and are translated here to English *COPD* Chronic obstructive pulmonary disease

The original question of occupation was the longest-term occupation (main occupation) each participant had. The occupational and socioeconomic coding (for NYK and SEI) of occupations was done for the present cohort during collection of the data in 1996-7 and were available in the current research data. From the two codes, a transformation could be made to obtain the ISCO-88 codes for the participants, and the Job-exposure matrix (JEM) used the ISCO-88 occupational coding as the basis of assigning occupational exposure. Both the SEI and NYK codes included participants who could not be classified in ISCO-88 such as students, long-term unemployed and pensioners without the data of occupation.

**Supplementary table 6.** Occupational coding definitions

| *SEI (Swedish Socio-economic index)^1^* | Two-to-three-digit coding from the original occupational title. Done previously by researchers in the 1990s based on the occupation title | *n = 6062* |
| --- | --- | --- |
| *NYK (Nordic Classification of Occupations)^2^* | Four-digit coding from the original occupational title. Done previously by researchers in the 1990s based on the occupation title | *n = 6062* |
| *ISCO-88 (International Standard Classification of Occupations, 1988 version)* | Transformation done in 2021, using the original SEI and NYK codings^3^ | *n = 5271* |

^1^ The Swedish Socio-Economic classification: Rationale and Fields of application
(<https://www.ergon-verlag.de/isko_ko/downloads/ic_15_1988_2_d.pdf>) (Last accessed 17.01.2022)

^2^https://www.scb.se/hitta-statistik/aldre-statistik/innehall/sveriges-officiella-statistik-sos/folk-och-bostadsrakningarna/1965-1990/1985/ (Last accessed 17.01.2022)

^3^Transformation form by Erik Bihagen: (<https://www.camsis.stir.ac.uk/occunits/distribution.html#Sweden>) (Last accessed 17.01.2022)

**Exposure data:**

In the study cohort, we had the original occupational classifications categorized by researchers in the 1990s with the Swedish socio-economic index (SEI) and Nordic classification of occupations (NYK). The two classifications were based on the title of the last long-term occupation as written by the participants in the 1996 postal questionnaire. From these two classifications we were able to approximate the international classification of occupations 1988 (ISCO88) class for the occupations using an existing category correspondence tool (Erik Bihagen, https://www.camsis.stir.ac.uk/occunits/distribution.html#Sweden).

Supplementary table has the definitions of the assessment of the Job-exposure Matrix values from the 1988 ISCO code.

**Supplementary Table 7.** The JEM values and the graded exposure variable

| JEM values: | Assessed from the ISCO-88 coding using the two first numbers | Biological dust | 0-2 (0 no exposure, 1 slight exposure, 2 strong exposure) |
| --- | --- | --- | --- |
|  |  | Mineral dust | 0-2 (0 no exposure, 1 slight exposure, 2 strong exposure) |
|  |  | Gases and fumes | 0-2 (0 no exposure, 1 slight exposure, 2 strong exposure) |
| Exposure: | Gathered from the JEM-values | None: | Value 0 in all three JEM categories |
|  |  | Intermediate: | Value 1 in any JEM category but no value 2 in any category |
|  |  | High: | Value 2 in any JEM category |

*JEM* job-exposure matrix; *ISCO-88* International Standard Classification of Occupations ver. 1988

The variable definitions for the study are shown in Supplementary table 8, which are derived from the postal questionnaire and the added mortality data.

**Supplementary Table 8.** Definitions of diagnosis, smoking, education status and mortality

| Diagnosis definitions: | *No diagnosis* | Answered no to both asthma and COPD diagnosed by a physician |
| --- | --- | --- |
|  | *Asthma* | Answered yes to asthma diagnosed by a physician |
|  | *COPD* | Answered yes to COPD diagnosed by a physician |
|  | *Co-existing Asthma and COPD* | Answered yes to both asthma and COPD diagnosed by a physician |
| Smoking definitions: | *Never-smoker* | Answered no to **both** *Current smoker* and *Ex-smoker* questions. |
|  | *Ever-smoker* | Answered yes to either *Current smoker* or *Ex-smoker* question. |
| Education status: | *Low* | Occupation titles requiring less than 2 years of post-comprehensive education as defined by SEI |
|  | *Intermediate* | Occupation titles requiring 2-5 years of post-comprehensive education as defined by SEI |
|  | *High* | Occupation titles requiring over 5-years of post-comprehensive education as defined by SEI |
| Mortality definitions: | *Respiratory mortality* | Respiratory disease (all ICD-10 J-codes) as the underlying cause of death |
|  | *Death associated with respiratory diseases* | Respiratory disease (all ICD-10 J-codes) either as the underlying cause or any contributing cause of death |

# Kaplan-Meier analysis

The Kaplan-Meier analysis was done for 50-years of age and older to have enough deaths in comparison to cases in the groups as well as trying to mitigate the effect of the age difference between the groups. Results are shown in Supplementary table 9.

**Supplementary table 9.** Mean survival for participants 50-years of age and older

| Overall mortality (age at 1996 > 50 years) | | | | | | | |
| --- | --- | --- | --- | --- | --- | --- | --- |
|  | *n*  cases | *n*  deaths | Mean survival time  (Years) | Standard deviation | 95% | CI | Pairwise comparisons |
|  |  |  |  |  |  |  | ***p*** |
| Asthma | 1537 | 591 |  |  |  |  |  |
| No diagnosis without exposure | 773 | 261 | 20.8 | 0.2 | 20.4 | . 21.2 | ref |
| No diagnosis with Intermediate exposure | 423 | 166 | 20.5 | 0.3 | 19.9 | - 21.0 | 0.075 |
| No diagnosis with High exposure | 245 | 129 | **18.7** | 0.4 | 17.9 | - 19.6 | **< 0.001** |
| Disease, No exposure | 52 | 15 | 21.9 | 0.6 | 20.8 | - 23.0 | 0.415 |
| Disease, Intermediate exposure | 29 | 11 | 19.9 | 1.2 | 17.5 | - 22.2 | 0.559 |
| Disease, High exposure | 15 | 9 | **18.2** | 1.7 | 15.0 | - 21.5 | **0.019** |
|  |  |  |  |  |  |  |  |
| COPD | 1508 | 592 |  |  |  |  |  |
| No diagnosis without exposure | 773 | 261 | 20.8 | 0.2 | 20.4 | . 21.2 | ref |
| No diagnosis with Intermediate exposure | 423 | 166 | 20.5 | 0.3 | 19.9 | - 21.0 | 0.075 |
| No diagnosis with High exposure | 245 | 129 | **18.7** | 0.4 | 17.9 | - 19.6 | **< 0.001** |
| Disease, No exposure | 29 | 15 | **19.7** | 1.1 | 17.5 | - 21.9 | **0.047** |
| Disease, Intermediate exposure | 21 | 10 | 19.0 | 1.4 | 16.1 | - 21.8 | 0.132 |
| Disease, High exposure | 17 | 11 | **15.9** | 1.9 | 12.3 | - 19.5 | **< 0.001** |
|  |  |  |  |  |  |  |  |
| Co-existing Asthma and COPD | 1484 | 586 |  |  |  |  |  |
| No diagnosis without exposure | 773 | 261 | 20.8 | 0.2 | 20.4 | . 21.2 | ref |
| No diagnosis with Intermediate exposure | 423 | 166 | 20.5 | 0.3 | 19.9 | - 21.0 | 0.075 |
| No diagnosis with High exposure | 245 | 129 | **18.7** | 0.4 | 17.9 | - 19.6 | **< 0.001** |
| Disease, No exposure | 16 | 10 | **17.8** | 1.9 | 14.1 | - 21.6 | **0.008** |
| Disease, Intermediate exposure | 12 | 9 | **15.6** | 2.0 | 11.9 | - 19.6 | **< 0.001** |
| Disease, High exposure | 15 | 11 | **14.1** | 1.9 | 10.3 | - 17.9 | **< 0.001** |

*p*-values are computed as factor level pairwise comparisons with Mantel-Cox

Statistically significant results are bolded

*COPD* chronic obstructive pulmonary disease; *Co-existing* both asthma and COPD diagnosed by a physician; *ref* reference group for pairwise comparisons
